# Context effects on processing widely deviant sounds in newborn infants

**DOI:** 10.3389/fpsyg.2013.00674

**Published:** 2013-09-27

**Authors:** Gábor P. Háden, Renáta Németh, Miklós Török, Sándor Drávucz, István Winkler

**Affiliations:** ^1^Research Program Language and Computation, Institute for Logic, Language and Computation, University of AmsterdamAmsterdam, Netherlands; ^2^Cognitive Science Center Amsterdam, University of AmsterdamAmsterdam, Netherlands; ^3^Department of Experimental Psychology, Institute of Cognitive Neuroscience and Psychology, Research Centre for Natural Sciences, Hungarian Academy of SciencesBudapest, Hungary; ^4^HonvédkórházBudapest, Hungary; ^5^Department of Cognitive and Neuropsychology, Institute of Psychology, University of SzegedSzeged, Hungary

**Keywords:** novelty detection, Event-Related Potentials (ERPs), auditory perception, context effects, human newborn

## Abstract

Detecting and orienting toward sounds carrying new information is a crucial feature of the human brain that supports adaptation to the environment. Rare, acoustically widely deviant sounds presented amongst frequent tones elicit large event-related brain potentials (ERPs) in neonates. Here we tested whether these discriminative ERP responses reflect only the activation of fresh afferent neuronal populations (i.e., neuronal circuits not affected by the tones) or they also index the processing of contextual mismatch between the rare and the frequent sounds. In two separate experiments, we presented sleeping newborns with 150 different environmental sounds and the same number of white noise bursts. Both sounds served either as deviants in an oddball paradigm with the frequent standard stimulus a tone (Novel/Noise deviant), or as the standard stimulus with the tone as deviant (Novel/Noise standard), or they were delivered alone with the same timing as the deviants in the oddball condition (Novel/Noise alone). Whereas the ERP responses to noise-deviants elicited similar responses as the same sound presented alone, the responses elicited by environmental sounds in the corresponding conditions morphologically differed from each other. Thus whereas the ERP response to the noise sounds can be explained by the different refractory state of stimulus-specific neuronal populations, the ERP response to environmental sounds indicated context-sensitive processing. These results provide evidence for an innate tendency of context-dependent auditory processing as well as a basis for the different developmental trajectories of processing acoustical deviance and contextual novelty.

## Introduction

Orienting toward new information is arguably the aspect of attention appearing earliest in life. Sounds widely differing from the preceding ones are amongst those most likely to initiate the orienting response (Sokolov, 1963), because they often accompany the arrival of a new object to the immediate environment or the emergence of an event that may have survival relevance. However, acoustic deviance is not the only informative aspect of a sound. The spectro-temporal makeup of a sound can reveal much about its source and/or the event that gave rise to it. Thus whereas acoustic deviation may serve as a trigger initiating a call for further processing (Öhman, 1979) the ensuing processing should depend on the relationship between the sound and the current context. For example, hearing a bird trill while walking in a forest may require less thought (processing) than the same sound encountered in one's bathroom. Some influential theories of perceptual object formation suggest a crucial role for contextual processing of sensory information (e.g., Ahissar and Hochstein, 2004; Bar, 2007). These theories assume that the perceiver has previously gathered a large amount of information about various contexts (scenes). This information must be learned during the course of life and thus cannot be available at birth. In accordance with this notion, most descriptions suggest that newborn infants are initially predisposed to orienting toward salient stimulus features and processes relating the incoming information to some representation of the context gradually appear during the first few month of life (Gomes et al., 2000). However, it is unlikely that each individual must discover the advantages of contextual processing for fast and robust discovery of objects in the environment. One should think that tendencies for contextual processing should already be present at birth (or even earlier), even if the effects on behavior cannot be observed (possibly due to the lack of extensive knowledge about various environments and the limited behavioral repertoire of neonates). Therefore, using a non-invasive brain measure event-related brain potentials (ERP), we tested the effects of acoustic context on the processing of widely deviant sounds.

In adults, sounds widely deviating from the preceding ones elicit a series of ERP responses (for a review, see Escera and Corral, 2003): a large fronto-central negativity (compared to regular sounds) peaking between 100 and 200 ms from stimulus onset, which is assumed to sum contributions from the auditory N1 and mismatch negativity (MMN) generators, followed by a positive component typically peaking between 200 and 350 ms (termed the P3a) and, if the listener has been engaged in some task for which the deviance was irrelevant, a later negativity peaking after 400 ms (the reorientation negativity; RON). The N1 is sensitive to spectral stimulus features (such as the frequency components present in a sound) and its amplitude increases when the time between successive presentations of the same spectral component is prolonged (Näätänen and Picton, 1987). The MMN component is elicited by violations of acoustic regularities, such as when a different sound is inserted into a repetitive sequence (Näätänen et al., 1978; Winkler, 2007). These ERP responses index two aspects of detecting acoustic change, with the N1-generating processes possibly involved in detecting the presence of new (or long heard) spectral features, whereas the processes assumed to give rise to MMN detecting deviations from the features of the preceding sound sequence. Traditional (deviant-minus-standard difference) estimates of the deviance-elicited differential response sum together the two contributions and it has long been debated in the adult MMN literature whether a genuine MMN component (Jacobsen and Schröger, 2001), a pure deviance-related process, exists (see, May and Tiitinen, 2010 vs. Näätänen et al., 2011; for a unifying view based on predictive coding, see Garrido et al., 2009). The P3a component is often interpreted as the ERP index of attentional capture (Escera et al., 2000; Friedman et al., 2001; Polich, 2007), although some recent results suggest that it may reflect evaluation of the relevance of the stimulus in a wider context (Horváth et al., 2008). Finally, RON is assumed to be an ERP sign of reorientation to the ongoing task following the distraction caused by the deviant stimulus (Schröger and Wolff, 1998; Berti and Schröger, 2001).

In one of their stimulus conditions, Kushnerenko and colleagues (2007) delivered to adults and newborn infants sequences in which a repetitive tone was occasionally exchanged for a white noise segment or an environmental sound (termed “novel” stimulus). They found that in newborn infants, these acoustically widely deviant sounds elicited a series of ERP responses that were somewhat similar to the adult ERPs: an early (150–220 ms) negativity (EN), a subsequent (250–300 ms) positivity (PC), and a late (400–600 ms) negativity (LN; for observations of similar responses, see Kushnerenko et al., 2002; Fellman et al., 2004; Sambeth et al., 2006; Háden et al., 2009). Although recorded in the context of acoustic deviance, the EN was primarily sensitive to the spectral richness of the sounds, whereas the PC was modulated by sound intensity. The N1 contribution to the adult ERP response is also sensitive to spectral features. On the other hand, in Kushnerenko et al.'s study, sound intensity did not elicit a large P3a in adults. The responses to noise segments and environmental sounds were morphologically quite similar, separately in the newborns and the adults. However, whereas noise segments elicited higher-amplitude responses for all three deflections in newborns, in adults, environmental sounds elicited higher amplitude N1/MMN responses than noise segments. These differences between the newborn and adult responses suggest that newborns and adults process partly different aspects of these sounds. The possible relation between these infantile ERP responses and the corresponding components in adults as well as the development of these responses during the first year of life are discussed in Kushnerenko et al. (2013).

Trainor and colleagues (Trainor et al., 2001, 2003; He et al., 2007, 2009) suggested that the refractoriness based detection of rare sounds develops earlier in life and it is shown by a slow positive shift in response to rare deviant sounds in young infants. Memory-based deviance detection would then develop somewhat later (4–6 months of life) and it is shown by negative ERP responses more similar to the adult MMN. However several neonate studies showed negative difference responses (e.g., Tanaka et al., 2001; Kushnerenko et al., 2002; Stefanics et al., 2009), while others obtained both positive and negative responses (e.g., Fellman et al., 2004; Kushnerenko et al., 2007; Háden et al., 2009). The issue of the polarity of deviance-related responses in young infants is not yet settled in the literature (for a discussion, see Kushnerenko et al., 2013).

The question addressed by the current study was whether or not noise segments and environmental sounds, which elicit similarly large reliable ERP responses when delivered amongst frequent tone stimuli (Sambeth et al., 2006; Kushnerenko et al., 2007), are processed in a context-dependent manner. Two alternative hypotheses were contrasted. (1) The responses observed for these sounds in the tonal context originate from neural generators sensitive to spectral richness with no influence of the surrounding tones. (2) A part of these responses reflects interactive processing (mismatch) of the tonal context and the noise/environmental sounds. For separating these alternatives, in separate experiments, novel sounds and white noise segments were, presented to newborn infants (a) in the context of pure tones (novel/noise-deviant condition), (b) as frequent sounds with pure tones as infrequent deviants (reversing the roles of the two types of sounds with respect to the novel/noise-deviant condition; novel/noise-standard condition), and (c) alone with the same temporal presentations schedule as in the novel/noise-deviant condition (novel/noise-alone condition; for a similar approach to this question, see Sams et al., 1985; Lounasmaa et al., 1989). If spectral richness alone determined the responses to the noise/environmental sounds then the responses to these sounds should only differ in amplitude across the three conditions: responses in the novel/noise-alone condition should be of similar, perhaps somewhat higher-amplitude than the ones obtained in the novel/noise-deviants condition and both significantly larger than those obtained in the novel/noise-standard condition. In contrast, if the processing of noise/environmental sounds was context-dependent, then morphologically different responses are expected to be elicited by these sounds in the three contexts.

## Experiment I

### Materials and methods

#### Participants

ERPs were recorded from 23 (6 male) healthy, full-term newborn infants during day 1–3 postpartum. The mean gestational age was 39.30 weeks (*SD* = 0.93), birth weight 3328 g (*SD* = 309.11), and the mean Apgar score 9/10. An additional 12 (4 male) infants' data were recorded, but discarded due to excessive electrical artifacts. Informed consent was obtained from one or both parents. The experiment was carried out in a dedicated experimental room at the maternity ward of Honvédkórház. The mother of the infant could opt to be present during the recording. The study was conducted in full accordance with the Helsinki Declaration and it was approved by the relevant ethics committees: ETT-TUKEB, Hungary, Institutional Review Board of the Institute of Cognitive Neuroscience and Psychology, Hungary.

#### Stimuli and procedure

Environmental (“novel”) sounds [150 unique sounds, also used in Otte et al. (2013)], such as dog barking, doorbell ringing, etc., and pure tones of 1000 Hz frequency were presented to sleeping newborn infants. All sounds were adjusted to 200 ms duration with 5–5 ms linear rise and fall times for the pure tones. Sound intensity was set to 70 dB SPL. Stimulus onset asynchrony (SOA) was 500 ms. Sounds were presented binaurally by E-Prime software (Psychology Software Tools, Inc., Pittsburgh, PA) through ER-1 headphones (Etymotic Research Inc., Elk Grove Village, IL, USA) connected via sound tubes to self-adhesive ear-couplers (Natus Medical Inc., San Carlos, CA, USA) placed over the infants' ears. In the novel-deviant condition, 15% of the stimuli were environmental sounds (each individual sound presented once) while 85% were pure tones (“tone standard”). They were delivered in a pseudorandom order enforcing at least 3 standards between successive novel sounds. The role of the novel sounds and tones were reversed in a separate stimulus block (novel-standard condition) with 85% environmental sounds, each sound repeated on average 5.6 times and 15%pure tones (“tone deviant”). In the novel-alone condition, novel sounds were delivered exactly as in the novel-deviant condition with the tones exchanged for silence of the same duration. Each condition was delivered in a separate stimulus block, 1000 stimuli in the novel-deviant and novel-standard conditions and 150 in the novel-alone condition. The order of the stimulus blocks was separately randomized for each infant.

#### EEG recording

EEG was recorded with Ag/AgCl electrodes attached to the scalp at the F3, Fz, F4, C3, Cz, C4, P3, Pz, and P4 locations according to the International 10–20 System. The common reference electrode was placed on the tip of the nose and the ground electrode on the forehead. Eye movements were monitored bipolarly between an electrode placed lateral to the outer canthus of left eye and Fp1. EEG was recorded with 24 bit resolution at a sampling rate of 1000 Hz by a direct-coupled amplifier (V-Amp, Brain Products, Munich, Germany). The signals were on-line low-pass filtered at 110 Hz.

#### Data analysis

EEG was filtered off-line between 1 and 30 Hz. For each stimulus, an epoch of 600 ms duration including a 100 ms pre-stimulus interval was extracted from the continuous EEG record. Epochs with a voltage change exceeding100 μV on any EEG or EOG channel were rejected from further analysis. The remaining epochs were baseline-corrected by the average voltage in the 100 ms pre-stimulus period and averaged separately for each stimulus type. Only infants with more than 75 artifact free trials (50% of all deviant/alone trials) were included in the analyses. The mean number of artifact-free trials per infant was 119 (85–149, *SD* = 16.51) for novel-deviant and 124 (84–149, *SD* = 17.56) for tone-deviant stimuli, 129 (82–150, *SD* = 14.56) for novel sounds delivered alone, and 701 (456–826, *SD* = 95.94) for novel-standard and 647 (472–759, *SD* = 81.97) for tone-standard stimuli.

For amplitude measurements, 80 ms wide time windows were selected based on the grand-averaged waveforms obtained at the Cz electrode. For the novel sounds, one window was centered on the negative peak in the 130–210 ms latency range of the response elicited by novels delivered alone and the other on the positive peak in the 233–313 ms latency range of the response to novel deviants. The effects of the stimulus conditions were analyzed with Three-Way repeated-measures analyses of variance (ANOVA: Stimulus condition [Deviant vs. Standard vs. Alone] × Frontality [Frontal vs. Central vs. Parietal] × Laterality [Left vs. Central vs. Right]).

A similar statistical analysis was conducted for the responses elicited by tones (ANOVA: Stimulus type [Deviant vs. Standard] × Frontality [Frontal vs. Central vs. Parietal] × Laterality [Left vs. Central vs. Right]. The 80 ms long time windows were selected from the grand-averaged deviant waveform at the Cz electrode. One window was centered on the negative peak in the 88–168 ms latency range and the other on the positive peak in the 236–316 ms latency range.

Greenhouse-Geisser correction of the degrees of freedom was applied where appropriate, and the ε correction factors as well as the η^2^_*p*_ effect sizes are given in the text. ANOVA results were further specified by Tukey's HSD *post-hoc* tests.

### Results

**Figure 1:**
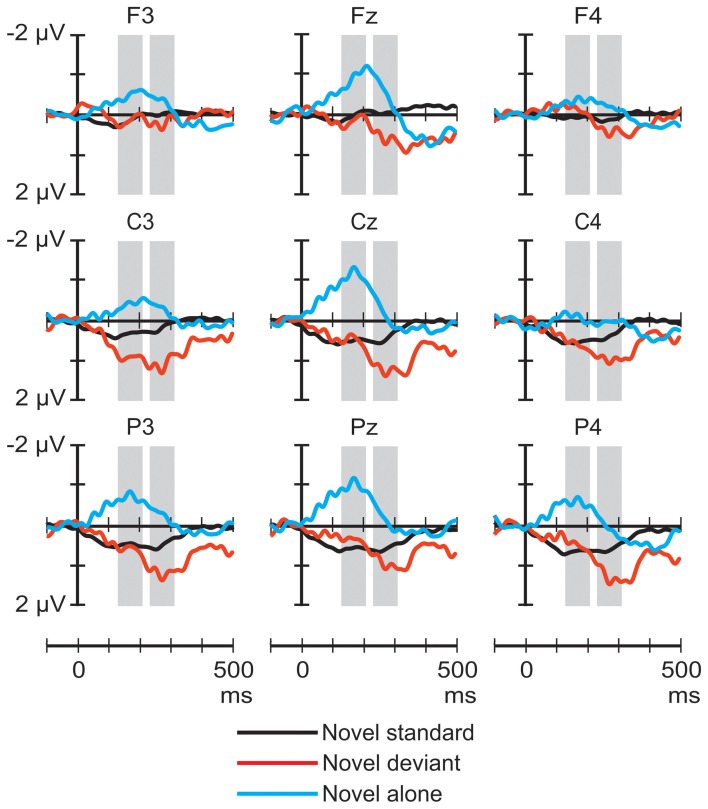
**Group-average (*n* = 23) ERP responses elicited by the novel sounds in the novel-deviant (red line), novel-standard (black), and novel-alone (blue) conditions at frontal, central, and parietal scalp locations.** Amplitude measurement windows are indicated by gray bars.

Figure 1 shows the ERP responses elicited by novel sounds in the three stimulus conditions. The ANOVA of the average amplitudes in the 130–210 ms latency range yielded a significant main effect of Stimulus condition [*F*_(2, 44)_ = 6.07, *p* < 0.01, η^2^_*p*_ = 0.22, ε = 0.77], Frontality [*F*_(2, 44)_ = 5.76, *p* < 0.02, η^2^_*p*_ = 0.21, ε = 0.74], and Laterality [*F*_(2, 44)_ = 4.04, *p* < 0.05, η^2^_*p*_ = 0.16], and a significant Stimulus condition × Laterality interaction [*F*_(4, 88)_ = 3.41, *p* < 0.05, ç_*p*2_ = 0.13, ε = 0.64]. *Post-hoc* tests indicated that the responses to novel sounds presented alone were significantly more negative than the responses to the novel standard (*p* < 0.02) or deviant (*p* < 0.01) stimuli. Frontal response amplitudes were significantly more negative than central ones (*p* < 0.01), and the negativity was significantly more pronounced at midline locations than on the right side of the head (*p* < 0.05). The interaction was explained by the significantly lower right than central or left response amplitudes for novel sounds presented alone (*p* < 0.01).

The ANOVA of the average amplitudes in the 233–313 ms latency range yielded a significant main effect of Stimulus condition [*F*_(2, 44)_ = 3.95, *p* < 0.05, η^2^_*p*_ = 0.15, ε = 0.68], and Frontality [*F*_(2, 44)_ = 8.11, *p* < 0.01, η^2^_*p*_ = 0.27, ε = 0.64]. *Post-hoc* tests showed that the responses to the novel deviant had significantly higher amplitudes than those elicited by the same sounds presented alone (*p* < 0.05), and the responses measured at frontal electrodes were significantly less positive than those measured at central (*p* < 0.01) and parietal (*p* < 0.01) locations.

**Figure 2:**
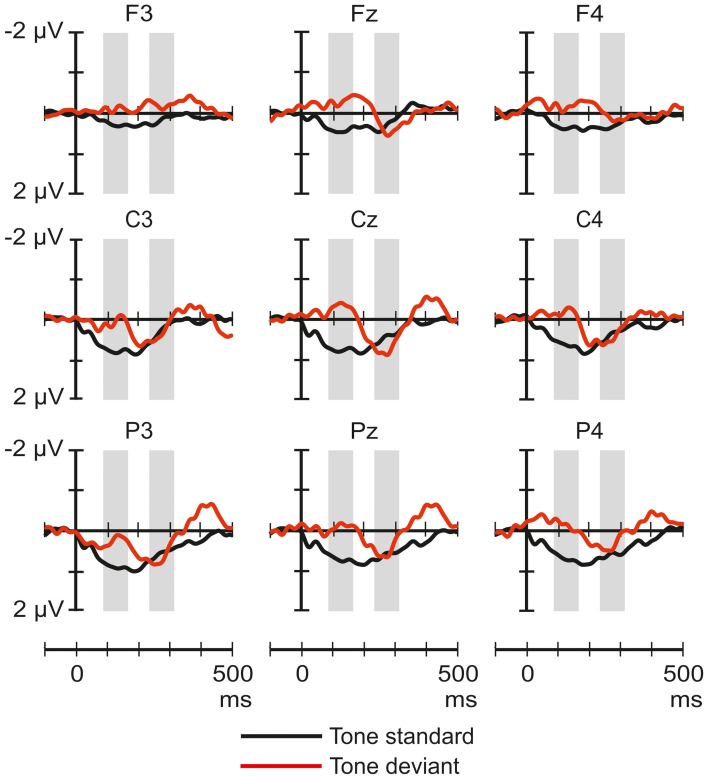
**Group-average (*n* = 23) ERP responses elicited by the pure tones in the novel-deviant (tone standard; black line), and novel-standard (tone deviant; red) conditions at frontal, central, and parietal scalp locations.** Amplitude measurement windows are indicated by gray bars.

Figure 2 shows the ERP responses elicited by tones in the novel-deviant and novel-standard conditions. The ANOVAs of the average amplitudes only yielded significant results in the 88–168 ms latency range. A significant main effect of Stimulus type [*F*_(1, 22)_ = 5.59, *p* < 0.05, η^2^_*p*_ = 0.2] and Frontality [*F*_(2, 44)_ = 3.8, *p* < 0.05, η^2^_*p*_ = 0.15, ε = 0.73] were observed. Deviant tones elicited more negative responses than the standards. *Post-hoc* test indicated a significantly more negative response at frontal than parietal sites (*p* < 0.05).

One possible confound is that the difference found between the stimulus conditions in Experiment 1 could have been caused by stimulus variation: Due to artifact rejection, responses in the different conditions could have been averaged from responses to acoustically different sounds. Further, unlike as deviants and alone, each novel sound appeared, on average, 5.6 times as standard. Therefore, the analysis was rerun by retaining only those responses, the corresponding ones (i.e., elicited by the same sound) for which were artifact free in all three conditions. Further, for the novel-standard condition, only the first artifact-free instance was included in the average ERP response. Thus the number of trials included in the average ERP responses became equal and the underlying unique sounds the same across the three conditions. In some infants, this led to utilizing fewer trials than what was required by the original criteria of 50% of all trials: on average 95 (65–123; *SD* = 15.53) trials were included in the average ERPs. The measurement windows were identical to those of the original analysis (130–210 ms and 233–313 ms, for the early and late windows, respectively).

For the early measurement window (130–210 ms), significant main effects of Stimulus condition [*F*_(2, 44)_ = 3.69, *p* < 0.05, η^2^ = 0.14, ε = 0.99] and Laterality [*F*_(2, 44)_ = 5.64, *p* = 0.01, η^2^ = 0.20, ε = 0.85] as well as a significant Stimulus condition × Laterality interaction [*F*_(4, 88)_ = 4.18, *p* < 0.01, η^2^ = 0.16, ε = 0.79] were found. *Post-hoc* Tukey-HSD test revealed that the Stimulus condition main effect was caused by more negative responses to Novel alone sounds than to Novel deviants, whereas the interaction was due to the Stimulus condition difference being smaller on the right than on the left side of the scalp. For the late measurement window (233–313 ms), only a main effect of Stimulus condition [*F*_(2, 44)_ = 3.27, *p* < 0.05, η^2^ = 0.12, ε = 0.99] was found. *Post-hoc* Tukey-HSD test showed that the Novel deviants elicited a more positive response compared to the Novel alone sounds.

## Experiment II

### Materials and methods

#### Participants

ERPs were recorded from 33 (18 male) healthy, full-term newborn infants during day 1–3 postpartum. The mean gestational age was 38.73 weeks (*SD* = 1.31), mean birth weight 3387 g (*SD* = 471), and the mean Apgar score was 9/10. An additional 17 (9 male) infants' data were recorded, but discarded due to excessive electrical artifacts. Ethical permissions and procedures were identical to those of Experiment I.

#### Stimuli and procedure

All procedures and stimuli were identical to Experiment I, except that environmental (novel) sounds were exchanged for white noise segments of 100 ms duration (including 5–5 ms linear rise and fall times). The three stimulus conditions are named “noise-deviant,” “noise-standard” and “noise-alone.”

#### EEG recording

Procedures and parameters were identical to Experiment I.

#### Data analysis

Filtering and artifact rejection was carried out as in Experiment I. The mean number of artifact-free trials per infant was 110 (75–143, *SD* = 18.91) for noise-deviant and 106 (80–141, *SD* = 16.18) for tone-deviant stimuli, 115 (78–143, *SD* = 16.40) for noise segments presented alone, and 643 (458–809, *SD* = 92.94) for noise-standard and 651 (431–798, *SD* = 101.78) for tone-standard stimuli.

Windows for average amplitude measurements were selected as in Experiment I. For responses to noise segments, one window was centered on the negative peak in the 118–198 ms latency range and the other on the positive peak in the 224–304 ms latency range. For responses to tones, one window was centered on the negative peak in the 20–100 ms latency range and the other on the positive peak in the 170–250 ms latency range. Statistical analyses were conducted as in Experiment I.

In addition, ERP responses elicited by pure tones as “deviants” (rare stimuli) were compared between Experiment I and II for assessing the contextual effects on processing the tones. Amplitudes for deviant tones were averaged from the 130–210 ms latency range. The effects were analyzed with mixed-design analysis of variance with “Experiment” as the grouping variable (ANOVA: Experiment [Noise vs. Novel] × Frontality [Frontal vs. Central vs. Parietal] × Laterality [Left vs. Central vs. Right]. All other procedures were identical to those set for the other statistical analyses.

### Results

**Figure 3:**
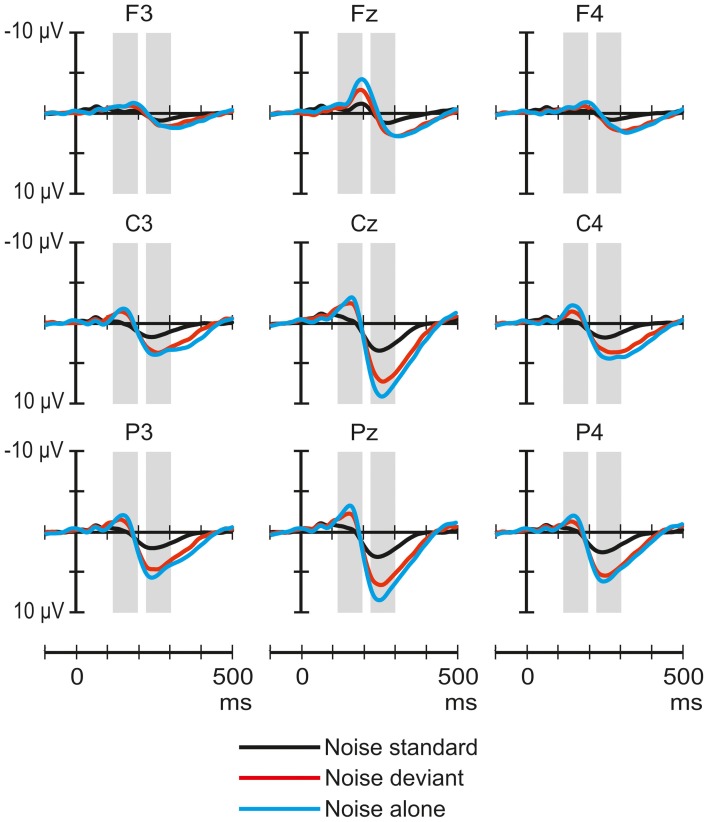
**Group-average (*n* = 33) ERP responses elicited by the noise segments in the noise-deviant (red line), noise-standard (black), and noise-alone (blue) conditions at frontal, central, and parietal scalp locations.** Amplitude measurement windows are indicated by gray bars.

Figure 3 shows the ERP responses elicited by noise segments in the three stimulus conditions. The ANOVA of the amplitudes in the 118–198 ms latency range of the responses to noise yielded a significant main effect of Stimulus condition [*F*_(2, 64)_ = 6.07, *p* < 0.01, η^2^_*p*_ = 0.16, ε = 0.8], and Laterality [*F*_(2, 64)_ = 12.15, *p* < 0.01, η^2^_*p*_ = 0.28]. *Post-hoc* tests showed a significant difference between the responses obtained in the noise-standard and noise-alone conditions (*p* < 0.01) and that responses measured at central electrode sites were significantly more negative than those measured either on the left (*p* < 0.01) or the right side (*p* < 0.01).

The ANOVA of the amplitudes in the 224–304 ms latency range yielded a significant main effect of Stimulus condition [*F*_(2, 64)_ = 19, *p* < 0.01, η^2^_*p*_ = 0.37], Frontality [*F*_(2, 64)_ = 32.13, *p* < 0.01, η^2^_*p*_ = 0.5, ε = 0.74], and Laterality [*F*_(2, 64)_ = 25.4, *p* < 0.01, η^2^_*p*_ = 0.44], as well as significant Stimulus condition × Frontality [*F*_(4, 128)_ = 16.75, *p* < 0.01, η^2^_*p*_ = 0.34, ε = 0.66], Stimulus condition × Laterality [*F*_(4, 128)_ = 8.44, *p* < 0.01, η^2^_*p*_ = 0.21], Frontality × Laterality [*F*_(4, 128)_ = 14.12, *p* < 0.01, η^2^_*p*_ = 0.31], and Stimulus condition × Frontality × Laterality interactions [*F*_(8, 256)_ = 6.62, *p* < 0.01, η^2^_*p*_ = 0.17, ε = 0.71]. *Post-hoc* tests showed that the responses in the noise-standard condition were significantly less positive than either in the noise-deviant (*p* < 0.01) or noise-alone condition (*p* < 0.01). Only the three-way interaction was analyzed further. This interaction was caused by the difference between noise standards and the other two responses being larger parietally than frontally (*p* < 0.01) and at the midline compared to the two sides (*p* < 0.01).

**Figure 4:**
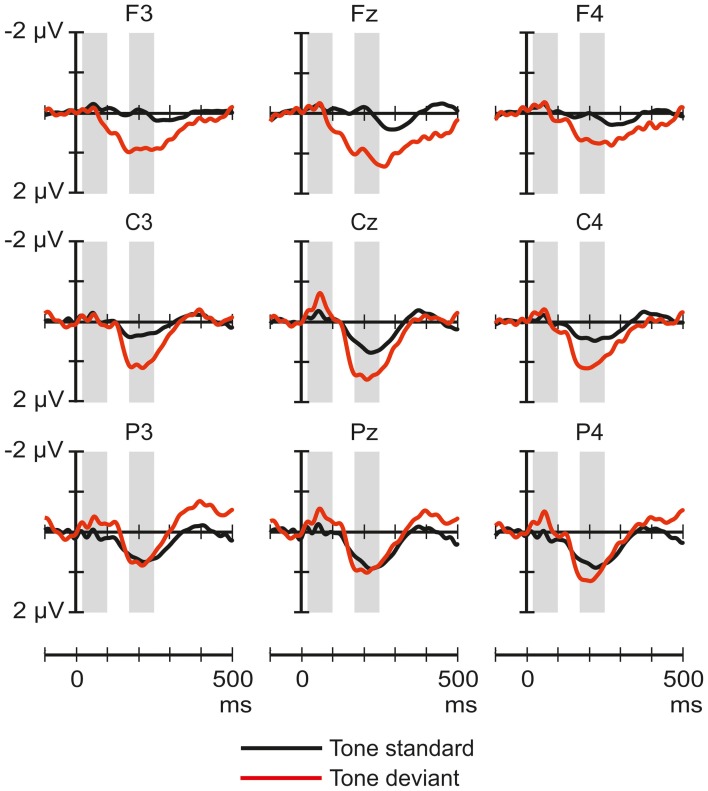
**Group-average (*n* = 33) ERP responses elicited by the pure tones in the noise-deviant (tone standard; black line) and noise-standard (tone deviant; red) conditions at frontal, central and parietal scalp locations.** Amplitude measurement windows are indicated by gray bars.

Figure 4 shows the ERP responses elicited by tones in the noise-deviant and noise-standard conditions. The ANOVA of the ERP amplitudes elicited by tones only yielded significant effects in the 170–250 ms window: a significant main effect of Stimulus type [*F*_(1, 32)_ = 7.53, *p* < 0.01, η^2^_*p*_ = 0.19], and Frontality [*F*_(2, 64)_ = 4.94, *p* < 0.05, η^2^_*p*_ = 0.13, ε = 0.71], and a significant Stimulus type × Frontality interaction [*F*_(2, 64)_ = 6.91, *p* < 0.01, η^2^_*p*_ = 0.18]. *Post-hoc* tests indicated that the standard tones elicited more positive responses than the deviants, especially at the frontal (*p* < 0.02) and central (*p* < 0.05) scalp locations.

**Figure 5:**
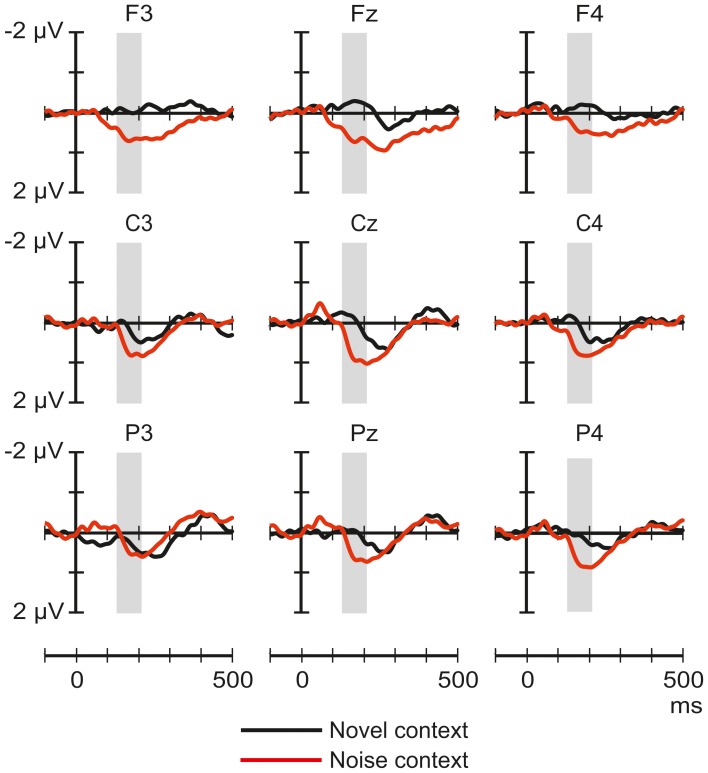
**Comparison between the group-averaged ERP responses elicited by the pure-tone deviants presented amongst frequent novel sounds (black line) and noise segments (red) at frontal, central, and parietal scalp locations.** The amplitude measurement window is indicated by the gray bar.

Figure 5 shows the ERP responses elicited by tones in the novel-deviant and noise-deviant conditions. The ANOVA comparing the tone deviant responses between the novel and the noise context yielded a significant main effect of Experiment [*F*_(1, 54)_ = 4.58, *p* < 0.05, η^2^_*p*_ = 0.08]: tone deviants presented amongst the noise segments elicited a more positive response than those presented amongst the novel stimuli.

## Discussion

The ERP responses elicited in sleeping newborn infants were similar to those obtained in previous experiments (Sambeth et al., 2006; Kushnerenko et al., 2007). Spectrally rich stimuli elicited higher-amplitude responses (much higher in the case of the noise segments) than pure tones. This result is compatible with the notion that these responses are at least partially governed by spectral content. In Experiment I, the rare environmental sounds delivered in the context of frequent pure tones elicited a positivity peaking between 200 and 400 ms over central and parietal sites (See Figure 1). In contrast, in the same latency range, the response to environmental sounds did not significantly differ whether the sounds were presented alone or with higher temporal density (in the novel-standard condition). However, when presented alone, environmental sounds elicited a broad negative waveform peaking at ca. 170 ms in the signals recorded over the midline. This waveform was not present in the responses elicited by the same sounds in the other two conditions (See Figure 3). As for the noise segments, the responses showed the same temporal pattern and similar scalp distribution in all three conditions of Experiment II: an early negative wave peaking at 158 ms followed by a large positive wave peaking at 264 ms. In the early time window, the amplitude of the response to the noise segments delivered alone was larger than that elicited in the noise-standard condition. In the late window, noise segments presented alone or as deviants elicited larger positive responses compared to when they were presented frequently while not differing significantly from each other. In both experiments, tone deviants elicited significantly different responses from tone standards and also from each other, when compared across the two experiments.

The component structure of the ERP responses elicited by environmental sounds grossly differed across the three conditions. This suggests that depending on the context, these sounds were processed qualitatively differently. Noise segments elicited responses with very similar component structure in the three conditions and the response amplitudes were modulated in accordance with the prediction based on the assumption that the underlying generators are not sensitive to the context. Instead, their activity was modulated by stimulus-specific refractoriness, most likely based on frequency-specific neural circuits. However, this interpretation is somewhat weakened by the significant effects of stimulus condition on the scalp distribution of the positive peak. Because the context (noise vs. environmental sounds) only affected the amplitude of the response to tone deviants (See Figure 5), but not the structure or scalp distribution of these ERP responses, this result can again be explained by stimulus-specific refractoriness. To summarize the findings, newborn infants process environmental sounds together with their context. The ERP responses obtained in our experiments for noise and tone sounds can be explained without assuming contextual processing. That is, these responses may reflect the refractoriness of some neuronal populations.

What makes environmental sounds different from noise segments that triggered contextual processing of the former but not of the latter? In general, unlike that of tone and noise segments, the spectral contents of environmental change in time (differently for each sound). The presence of dynamic spectral changes could thus serve to separate the environmental sounds from the other two types of sounds even without previous experience with these sounds. Kushnerenko et al. (2007) suggested that the newborn brain uses simple perceptual categories based on basic auditory features. Based on this view, newborn infants may categorize sounds by the presence or absence of dynamic spectral changes. However, because each environmental sound had a unique temporal structure, they may have required more advanced processing than simpler sounds and thus activated systems responsible for contextual processing. That is by lacking semantic categories, newborns probably could not form a category for the environmental sounds as adults and young children do (Wetzel et al., 2009).One may speculate that detecting the presence of dynamic spectral changes may be useful for infants to separate sounds with potential survival relevance (such as animal sounds) from ones that are less likely to have such relevance (such as the sound of the wind).

Because the environmental sounds were unique, whereas the noise segments did not differ from each other, the latter appeared with somewhat higher probability than the former. Thus an alternative explanation could suggest that the difference in probability led to contextual processing for novel sound but not for noise segments. This explanation assumes that the neonate brain formed memory traces of the noise/novel sounds and detected the repetition of the noise segments. Results in adults, showing that in a passive situation, only the most frequent sound acts as the “standard” for MMN elicitation argues against this assumption. Sussman and colleagues (2003) showed that in a sequence with a frequent (83.5%), a medium frequent (14.5%), and an infrequent tone (2.5%), the infrequent tone elicited the MMN only with respect to the frequent tone, but not with respect to the medium frequent tone, even though the medium frequent tone also elicited MMN with respect to the frequent tone and the ratio between the sequential probabilities of the frequent and medium frequent tones was approximately the same as that between the medium frequent and the infrequent tone. Thus it appears that in adults, the memory trace underlying pre-attentive deviance detection is only formed for the frequent, but not for the infrequent sound within the sequence.

A further possible confound is that the ratio between male and female infants was different across the two experiments. There is evidence that phoneme processing differs between 4 week old male and female infants (Friederici et al., 2008; Mueller et al., 2012). It is possible that developmental sex differences extend to the processing of complex sounds in general, which could then have biased the current results. However, previous studies testing similar stimuli in groups of sufficient size to check sex differences in processing rare novel sounds and noise segments found no effect of sex (e.g., Otte et al., 2013).

We also found some differences between the responses elicited in the current experiments and the few previous studies presenting similar stimuli under similar conditions. In Kushnerenko et al.'s (2007) study, the responses elicited by both environmental and noise sounds followed the EN-PC-LN structure, with peak amplitudes close to or above 5 μV. The late negativity was absent from the current traces, probably due to the faster presentation rate (500 in the current paradigm vs. 800 ms SOA in Kushenrenko et al.'s study). Despite this difference, the responses to noise segments had very similar time course and amplitudes across these two studies. This demonstrates that noise segments elicit robust responses which are replicable in different laboratories and they are also fairly similar across individuals. In contrast, the current responses to environmental sounds showed no EN and the PC amplitude was lower than that elicited by the noise segments or the environmental sounds tested by Kushnerenko and colleagues. The current novel responses were much smaller in amplitude and appeared to be less dominated by spectral richness, in general. These differences may be due to the difference in presentation rate and/or that a different set of environmental sounds was delivered. The latter may have been a factor especially if the current set of environmental sounds included larger acoustic variability than that of Kushnerenko and colleagues. Despite these differences the current results and those of Kushnerenko and colleagues do not contradict each other; however the differences across experiments obtained for the responses elicited by environmental sounds suggest that these sounds evoke much less stable responses than noise segments. Otte et al. (2013) presented 2 months old infants with the same environmental sounds as were presented to newborns in the current study. Also the SOA was identical between the two experiments. The response observed in 2-month olds was, however, somewhat different from the current responses, consisting of a large positive waveform peaking ca. 100 ms earlier than the PC elicited in the current study. This developmental change of the ERP responses is compatible with previous findings (Kushnerenko et al., 2002; He et al., 2009; for a review and discussion, see Kushnerenko et al., 2013).

In summary we have shown that neonates can process sounds contextually. Thus the prerequisites of context-based perceptual object formation are present already at birth. Further, similarly to adults (see Kushnerenko et al., 2007), newborn infants process environmental sounds differently from noise segments and tones.

### Conflict of interest statement

The authors declare that the research was conducted in the absence of any commercial or financial relationships that could be construed as a potential conflict of interest.
